# Prognostic value of the systemic inflammation response index (SIRI) in glioma: A meta-analysis

**DOI:** 10.1371/journal.pone.0362113

**Published:** 2026-11-02

**Authors:** Lixia Guo, Xudong Cao, Lijun Yang, Xiaoyan Zhu, Lan Liu, Huandong Liu

**Affiliations:** 1 Department of Medical College, Xizang University, Lhasa Tibet, China; 2 Department of Neurosurgery, People’s Hospital of Xizang Autonomous Region, Lhasa, Xizang Autonomous Region, China; Xiangya Hospital Central South University, CHINA

## Abstract

**Objective:**

To systematically evaluate the predictive value of systemic inflammation response index (SIRI) for the prognosis of glioma patients.

**Methods:**

PubMed, Cochrane Library, EMBASE, and Web of Science databases were searched to collect literature on the relationship between SIRI and glioma prognosis from database inception to July 2026. The primary outcome measures were overall survival (OS) and progression-free survival (PFS). STATA 15.0 and Review Manager 5.4 software were used, and random-effects models were employed with pooled hazard ratios (HR) and their 95% confidence intervals (CI) to assess the prognostic value of SIRI. Subgroup analyses, sensitivity analyses, and publication bias assessments were performed.

**Results:**

Eight retrospective cohort studies involving 1778 glioma patients were included, forming 12 comparison groups for OS analysis and 4 comparison groups for PFS analysis. Meta-analysis results showed that a higher SIRI was significantly associated with poorer OS (pooled HR = 1.79, 95% CI: 1.34–2.38, P < 0.0001) and PFS (pooled HR = 1.46, 95% CI: 1.18–1.81, P = 0.0005) in glioma patients. Subgroup analysis showed that the prognostic value of SIRI for OS was not significant in the European population subgroup, suggesting that the association may be modulated by geographic region. Sensitivity analysis confirmed the robustness of the results. Egger’s test indicated publication bias in the OS analysis (P = 0.0001), but no significant bias was found in the PFS analysis.

**Conclusion:**

This meta-analysis suggests that elevated SIRI is associated with poorer OS and PFS in glioma patients, although this association was not consistently observed across all subgroups, particularly in European populations. These findings highlight the need for further validation with standardized cut-off values in diverse populations.

## 1. Introduction

The most prevalent primary malignant brain tumor among adults, glioma encompasses both low-grade and high-grade types [1,2]. More than half of glioma patients are diagnosed with high-grade gliomas after postoperative pathology, which are characterized by high invasiveness, frequent recurrence, and poor prognosis [1,2]. Among primary brain tumors, high-grade gliomas, particularly glioblastoma, are both the most prevalent and aggressive. Although highly invasive, they remain confined to the central nervous system and do not metastasize [3]. Glioblastoma is marked by a high mortality rate and poor prognosis [1]. It is known for its ability to infiltrate multiple brain lobes and deep structures, complicating treatment and contributing to its aggressive nature [2]. The incidence rate is estimated at 3.19 per 100,000 people annually [4], with a 5-year survival rate of less than 10% [5,6]. Exploring biomarkers that enable rapid and convenient assessment of prognosis and treatment outcomes holds substantial clinical value.

In clinical practice, glioma prognosis is primarily predicted based on age, tumor grade, and molecular characteristics [7]. However, accurate prediction remains challenging, highlighting the need for better biomarkers to predict patient outcomes. Inflammation, a hallmark of tumor initiation and progression, plays a critical role in tumor growth, invasion, angiogenesis, and metastasis [8,9]. SIRI is a simple, cost-effective, and practical inflammation marker, calculated as: neutrophil count × monocyte count/lymphocyte count. Based on neutrophils, monocytes, and lymphocytes, it offers a comprehensive assessment of the relationship between anti-tumor immune effects and inflammation. Its prognostic value has been confirmed in various malignancies [10–12]. Jarmuzek et al. found that SIRI levels were significantly elevated in the glioma patient population, with higher mortality rates in WHO grade 4 gliomas among patients with high SIRI [13]. A retrospective analysis by Qian He [14] of clinical data from high-grade glioma patients who underwent surgery at the First Affiliated Hospital of Chongqing Medical University between December 2013 and December 2019 found that SIRI has prognostic value in high-grade gliomas.

The prognosis of glioma patients varies greatly, and accurate risk stratification is crucial for developing individualized follow-up intensity and treatment strategies. High-risk patients may benefit from closer imaging monitoring or more aggressive adjuvant therapy, while low-risk patients can avoid unnecessary overtreatment. Currently, clinical prognostic assessment mainly relies on age, tumor grade, and molecular markers (such as IDH mutation status and 1p/19q co-deletion), but these indicators cannot fully capture the dynamic changes in the host’s immune-inflammatory state [15]. The tumor microenvironment of gliomas is highly dependent on inflammation and immune escape mechanisms. SIRI, as an indicator reflecting systemic immune-inflammatory balance, holds promise for providing supplementary prognostic information orthogonal to existing molecular markers, thereby filling the gaps in the current risk stratification system [16].

It is worth noting that a similar meta-analysis published by Jiang et al. in 2025 also reported that elevated SIRI was significantly associated with poor OS and PFS in glioma patients, which is consistent with the core conclusions of this study and indirectly verifies the robustness of SIRI as a prognostic biomarker [17]. However, the search cutoff date for that study was relatively early, and it did not include the latest cohort data. Therefore, the purpose of this study is to include the latest clinical research based on previous meta-analyses to further validate the association between SIRI and glioma prognosis.

## 2. Materials and methods

### 2.1. Search strategy

This study adheres to the PRISMA 2020 statement [18] and was registered in PROSPERO (CRD420251072220). PubMed, Cochrane Library, EMBASE and Web of Science were conducted to search literature. The search covered publications up to July 2026, using the keywords “systemic inflammation response index” and “glioma.” The PubMed search query is as follows: (“Glioma”[Mesh]) OR ((((((((Glioblastoma) OR (Gliomas)) OR (Glial Cell Tumors)) OR (Glial Cell Tumor)) OR (Mixed Glioma)) OR (Mixed Gliomas)) OR (Malignant Glioma)) OR (Malignant Gliomas))) AND ((systemic inflammation response index) OR (SIRI)). The details of searching strategy are listed in S1 Table. Additionally, the reference lists were manually reviewed. Two researchers conducted the literature search and selection process independently. Discrepancies were resolved by discussion.

### 2.2. Study participants

Inclusion criteria:

(1) Studies on the prognostic value of baseline SIRI in gliomas (glioblastoma, high-grade glioma, and gliomas);
(2) Histologically confirmed glioma diagnosis via biopsy;
(3) Focus on evaluating the correlation between baseline SIRI and survival outcomes, such as PFS and OS;
(4) Studies providing hazard ratios (HR) and 95% confidence intervals (CI), either directly extracted or calculated from available data;
(5) Studies dividing cases into high baseline SIRI and low baseline SIRI groups;
(6) Cohort studies that are fully published.

Exclusion Criteria:

(1) Reviews, commentaries, conference abstracts, case reports, and letters;
(2) Studies lacking sufficient data to calculate HR and 95% CI;
(3) Studies without survival data;
(4) Studies with overlapping or duplicate data.

Titles and abstracts were initially reviewed, and full text were downloaded for further screening. Finally, only the studies that meet the inclusion criteria can be reserved. The two researchers reached an agreement through discussion.

### 2.3. Data extraction

First author’s name, publication year, country (study location), study type, sample size, patient age, study duration, threshold values, follow-up time, and HR for OS and PFS with 95% CI were extracted by two researchers independently.

### 2.4. Quality assessment

Newcastle-Ottawa Scale (NOS) was conducted to assess the literature quality, which allocates up to nine points across three parameters, selection, comparability, and outcome. Score ranges from 0 to 9 [19]. Studies scoring 7–9 were rated high-quality [20].

### 2.5. Statistical analysis

Combined HR and 95% CI were calculated to evaluate the prognostic value of SIRI in patients with glioma. Cochran’s Q test and Higgins’ I^2^ statistic were used to assess heterogeneity. Significant heterogeneity was indicated by I^2^ > 50% or P < 0.1. Random-effect models were employed. Sensitivity and subgroup analyses were used to explore the result stability and heterogeneity sources. Publication bias was examined with funnel plots and Egger’s test, and P < 0.05 denoted significance. Analyses were performed in STATA 15.0 and Review Manager 5.4.

## 3. Results

### 3.1. Literature search results

Ninety articles were retrieved from four databases. Thirty articles were removed due to duplication. Titles and abstracts were reviewed, and 45 articles did not meet the inclusion criteria. Nine articles were excluded after full-text reading. Ultimately, eight studies [13,14,21–26] were included. The flowchart was shown in Fig 1. One study [21] included two comparison groups, while another [27] included four, totaling ten comparison groups. All studies were retrospective cohort studies between 2021 and 2026. All participants were glioma patients, classified into high SIRI and low SIRI groups. SIRI was measured at the time of admission in all six studies. Ten comparison groups assessed the prognostic significance of SIRI for OS, and three comparison groups evaluated its prognostic value for PFS. Table 1 depicted the characteristics of studies.

**Table 1:** Baseline characteristics of included studies.

Authors	Study design	Types of cancer	Patients (n)	Gender		Age	Cut-off
				Male	Female		
Balci Topuz 2026	Retrospective cohort	Glioblastoma	92	47	45	58	2.35
Hou 2026	Retrospective cohort	Glioblastoma	143	78	65	60	1.45
Wang,Z. H. 2022a	Retrospective cohort	Glioblastoma	291	186	105	53.7 ± 14.2	1.26
Wang,Z.H. 2022b	Retrospective cohort	Glioblastoma	196	147	49	54.2 ± 13.8	1.26
He,Q.2021a	Retrospective cohort	High-grade glioma	62	30	32	50.44 ± 14.642	1.26
He,Q.2021b	Retrospective cohort	High-grade glioma	48	22	24	52.04 ± 14.244	1.26
He,Q.2021c	Retrospective cohort	High-grade glioma	43	27	16	48.56 ± 11.189	1.26
He,Q.2021d	Retrospective cohort	High-grade glioma	30	16	12	47.36 ± 10.393	1.26
Shi, X. H.2022	Retrospective cohort	Glioblastoma	232	127	105	NA	1.78
Jarmuzek, P.2022	Retrospective cohort	Glioblastoma	358	195	163	59.9 ± 13.5	NA
Aydin, A. A.2024	Retrospective cohort	Gliomas	198	114	84	60 (25–86)	NA
Clavreul,Anne2021	Retrospective cohort	Glioblastoma	85	65	20	60 (36–81)	2.55

**Fig 1:**
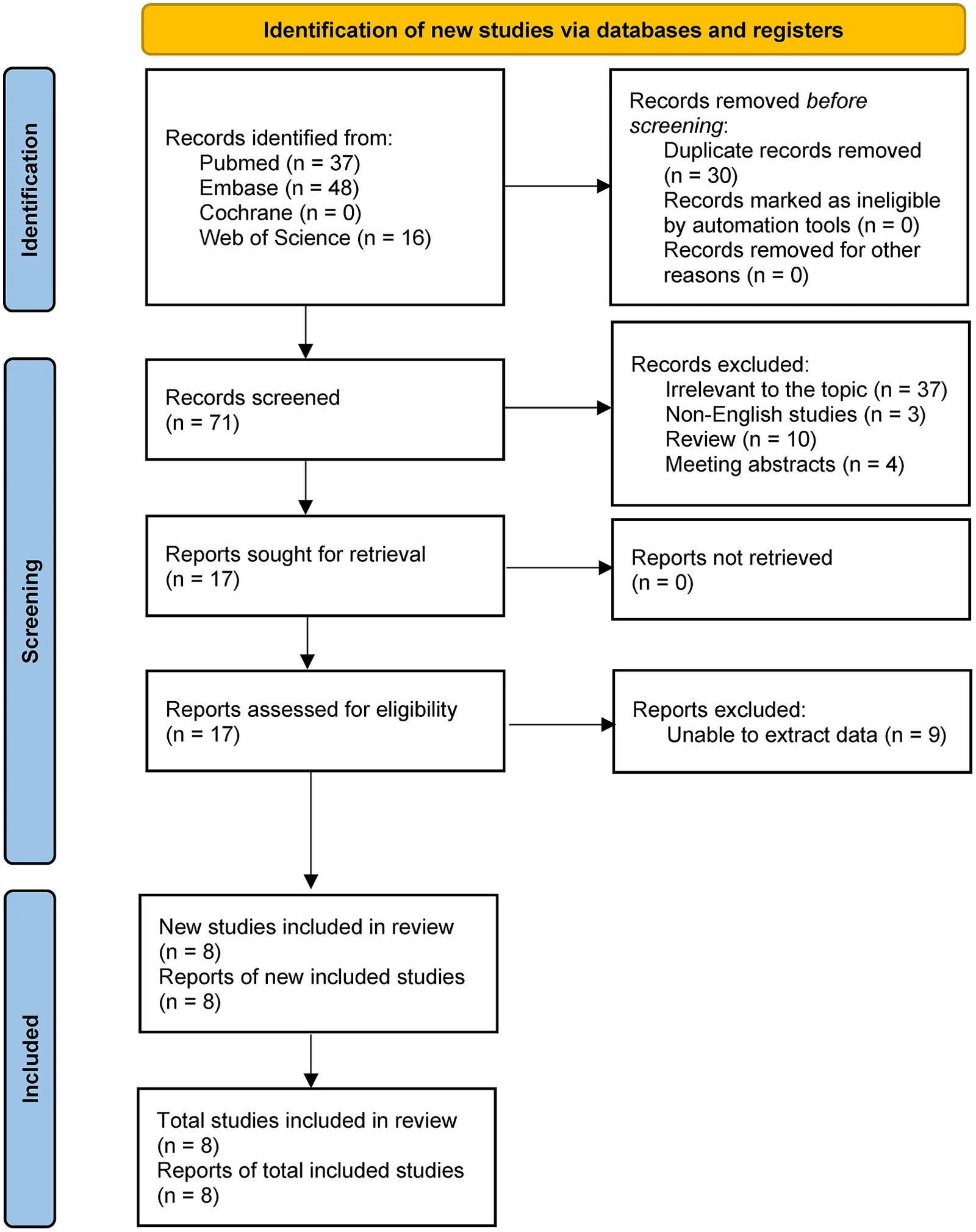
Literature screening flow chart.

### 3.2. Evaluation of literature quality

The 6 literatures scored 7–8 points, all of which were high-quality research papers (Table 2).

**Table 2:** Quality evaluation of the eligible studies with Newcastle–Ottawa scale.

Study	Selection				Comparability		Outcome		
	Representative-ness	Selection of non-exposed	Ascertainment of exposure	Outcome not present at start	Comparability on most important factors	Comparability on other risk factors	Assessment of outcome	Long enough follow-up (median≥1 year)	Adequacy (completeness) of follow-up
Wang, Z. H. et al.	*	*	*	*	–	–	*	*	*
Balci Topuz et al.	*	*	*	*	*	–	*	*	*
Hou et al.	*	*	*	*	–	–	*	*	*
He, Q.et al.	*	*	*	*	*	–	*	*	*
Shi, X. H. et al.	*	*	*	*	*	–	*	*	*
Jarmuzek, P. et al.	*	*	*	*	*	–	*	*	*
Aydin, A. A. et al.	*	*	*	*	*	–	*	*	*
Clavreul, Anne. et al.	*	*	*	*	*	–	*	*	*

*indicates criterion met; – indicates significant of criterion not met.

### 3.3. Meta-analysis results

#### 3.3.1. Prognostic value of SIRI for OS.

This study included 12 comparison groups involving 1778 patients, investigating the relationship between SIRI and OS. All 12 comparison groups provided baseline SIRI data. The forest plot shows that high SIRI values are significantly associated with shortened OS in glioma patients (HR = 1.79, 95% CI: 1.34–2.38, I^2^ = 86%) (Fig 2A). Additionally, Egger’s test (P = 0.0001) and funnel plot indicated publication bias (S1A Fig).

**Fig 2:**
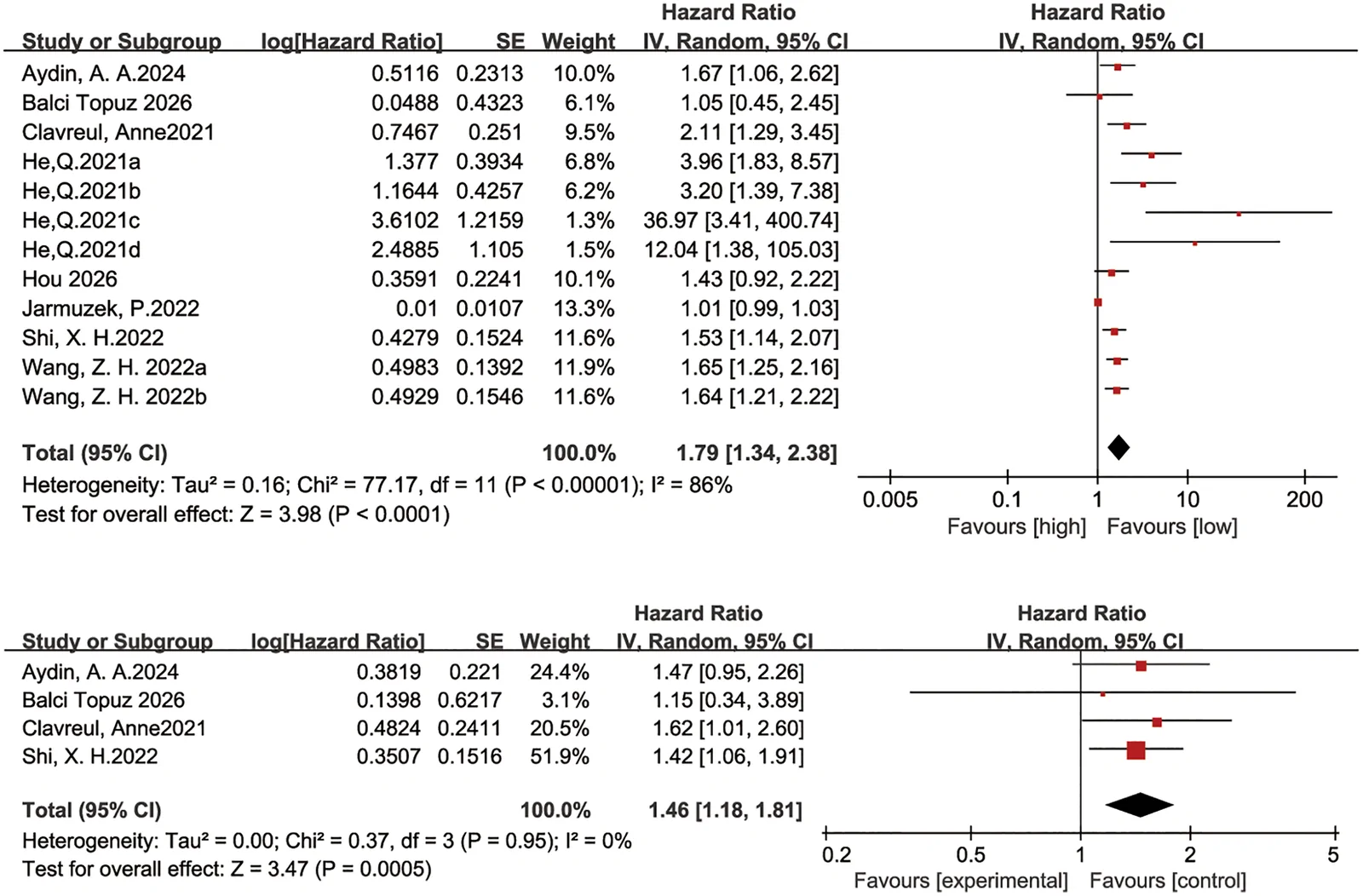
Mortality forest map. (A) Overall survival (B) progress free survival.

#### 3.3.2. Prognostic value of SIRI for PFS.

Four comparison groups, involving 607 patients, were analyzed to explore the link between SIRI and PFS. All comparison groups provided baseline SIRI data. The forest plot shows that high SIRI values are significantly linked with shortened PFS in glioma patients (HR = 1.46, 95% CI: 1.18–1.81, I^2^ = 0%) (Fig 2B). Egger’s test (P = 0.729) and funnel plot indicated no publication bias (S1B Fig).

### 3.4. Sensitivity analysis

SIRI remained stable in predicting prognostic outcomes, including OS and PFS in patients with glioma. The results remained consistent even after excluding any single study, unaffected by outliers. The sensitivity analysis results are shown in S2 Fig.

### 3.5. Subgroup analysis

The subgroup analyses in this study are descriptive and exploratory in nature; no formal interaction tests for subgroup differences or meta-regressions were performed. Consequently, any observed differences between groups should be viewed solely as leads for generating hypotheses. Subgroup analysis showed that both unclassified glioma (HR = 3.75) and glioblastoma (HR = 1.50) subgroups demonstrated significant prognostic value, but the latter exhibited extremely high heterogeneity (I^2^ = 89%). Notably, when stratified by SIRI cutoff values, regardless of whether a cutoff value of ≥1.5 or <1.5 was used, a high SIRI was significantly associated with poorer OS; however, the point estimates differed between the two groups. Given the substantial variation in the cutoff values selected across the included studies, this difference in numerical values is reported for descriptive purposes only and does not imply effect modification. Furthermore, geographical analysis showed significant results in the Asian population subgroup (n = 10) (HR = 2.00, I^2^ = 58%), while the European population subgroup did not reach statistical significance due to its small sample size (n = 2) (HR = 1.40, P = 0.36, I^2^ = 88%). Details of subgroup analysis are shown in Table 3.

**Table 3:** Detailed results of the subgroup analysis.

Subgroup	OS			
	Study group	HR [95%CI]	P value	I^2^
Total	12	1.79 [1.34–2.38]	<0.0001	86%
Type of tumor				
Unclassified glioma	6	3.75[1.77–7.94]	0.0006	66%
Glioblastoma	6	1.50[1.09–2.06]	0.01	89%
SIRI cut-off				
≥ 1.5	3	1.69 [1.27–2.25]	0.0003	15%
< 1.5	7	2.56 [1.61–4.05]	<0.0001	68%
Region				
Asia	10	2.00 [1.50–2.65]	<0.00001	58%
Europe	2	1.40 [0.68–2.87]	0.36	88%

## 4. Discussion

The results of this meta-analysis demonstrated that an elevated baseline SIRI was significantly associated with poorer OS in patients with glioma. This association was primarily driven by pooled evidence from studies focusing on high-grade gliomas, particularly glioblastoma, thereby supporting the prognostic relevance of SIRI in this aggressive tumor subtype characterized by rapid progression and unfavorable outcomes. Glioblastoma is known to exhibit a profoundly immunosuppressive tumor microenvironment, in which malignant progression is closely linked to systemic inflammatory responses, rendering composite inflammatory indices such as SIRI potentially more sensitive indicators of tumor burden and host immune status [17,28]. Nevertheless, it should be explicitly acknowledged that the included studies encompassed gliomas of varying histological grades and molecular subtypes and were subject to substantial clinical and methodological heterogeneity. Accordingly, the present findings should be interpreted as an exploratory assessment of inflammation-based prognostic modeling across the broader glioma spectrum, with the principal evidentiary support predominantly derived from high-grade gliomas. Caution is therefore warranted when extrapolating the prognostic utility of SIRI across heterogeneous glioma populations.

The pronounced heterogeneity of gliomas, particularly the fundamental differences between low-grade and high-grade tumors in disease trajectory, treatment strategies, and prognosis, substantially influences the interpretation of the prognostic value of SIRI [17]. High-grade gliomas, such as glioblastoma, are characterized by rapid growth, prominent inflammatory cell infiltration within the tumor microenvironment, and a more pronounced systemic inflammatory response, rendering inflammation-based indices like SIRI more sensitive to active disease burden [14,22]. In contrast, low-grade gliomas typically follow an indolent clinical course that may extend over several years or even decades, with a relatively quiescent tumor microenvironment and weaker systemic inflammatory responses that are more susceptible to confounding by long-term non-tumor-related factors, including intercurrent infections and chronic comorbidities [29–31]. Therefore, the available evidence suggests that the association between SIRI and survival may be more pronounced in high-grade glioma populations, although this observation is based on subgroup analyses with limited statistical power and should be considered hypothesis-generating rather than conclusive. Definitive demonstration of differential prognostic value across histological grades would require formal interaction testing in individual patient data meta-analyses with multivariable adjustment for key confounders.

Compared to recently published meta-analyses, this study offers incremental contributions in several aspects [17]. First, based on peer review comments, we expanded the search period to include cohort studies published up to 2026, updating the search cutoff date to July 2026 and incorporating updated literature evidence. Second, our subgroup analysis revealed effects of SIRI cutoff values (≥1.5 vs < 1.5) and geographic region (Europe vs Asia) on prognostic value. However, differences related to geographic regions require validation in larger samples, and the variations in pooled effects observed across different cut-off-based stratifications should also be considered descriptive findings. The consistency of our meta-analysis with previously published meta-analyses in core conclusions further supports the potential of SIRI as a prognostic marker for gliomas, while the additional findings of this study provide more nuanced clues for future research directions in this field.

Hematological markers are a key focus in clinical cancer research [32,33]. due to their easy accessibility and non-invasive nature, which have facilitated large-scale prospective and retrospective studies. The tumor microenvironment is closely linked to inflammatory cascades, including platelet activation and coagulation function activation, which trigger the release of inflammatory cytokines [9]. Several peripheral blood-related inflammatory markers are used clinically to assess and quantify systemic inflammation. Key indicators include the NLR, derived NLR, LMR, and PLR. These markers have demonstrated strong prognostic and predictive value in various cancers [34–37]. Compared to the afore mentioned markers, the SIRI is a novel prognostic indicator. The malignant progression of gliomas is also linked to systemic inflammation. Preoperative hematological marker levels vary across glioma grades and have predictive value [38].

SIRI, as a novel prognostic indicator, was initially identified in pancreatic cancer patients [39]. SIRI may influence the prognosis of malignant tumor patients through various mechanisms. Neutrophils contribute to the formation of the inflammatory microenvironment, suppress lymphocyte activity, inhibit T cell responses, and stimulate angiogenesis, all of which facilitate tumor growth and metastasis, exerting an immunosuppressive effect [40–43]. Lymphocytes play a crucial role in the body’s anti-tumor immune response by inhibiting tumor proliferation and metastasis, thereby exerting anti-tumor effects [44,45]. Monocytes, particularly tumor-associated macrophages (TAMs), can promote tumor growth and metastasis, and induce macrophages to enhance angiogenesis through the expression of CXCL1 and CXCL8 [46,47].

Despite its insights, the meta-analysis has limitations. An important limitation is the potential for unmeasured confounding factors, including perioperative corticosteroid use, tumor size and location, and concomitant medications, which may influence both SIRI levels and patient outcomes. Besides, an inherent limitation of this meta-analysis is that all included studies dichotomized SIRI using various cut-off values, which may lead to information loss and reduced statistical efficiency. Ideally, treating SIRI as a continuous variable would preserve more prognostic information. However, the lack of individual patient data precluded such an approach. Future studies should consider reporting effect estimates across the full range of SIRI values or adopting standardized cut-off determination methods to facilitate cross-study comparability. The inability to adjust for these confounders in the included studies precludes causal inference and underscores the need for prospective studies with comprehensive covariate ascertainment. The predominance of Asian, particularly Chinese, cohorts confine generalizability to Europe, Africa, the Americas or other regions; external validation is required. Retrospective designs predominated, carrying inherent confounding. Reported SIRI thresholds varied widely (1.26–2.55), contributing to heterogeneity; a uniform cutoff should be established. Finally, although all included studies scored 7–8 on the NOS, indicating acceptable methodological quality, this should not be interpreted as absence of bias. The NOS has limited discriminatory power for retrospective cohort studies, particularly in the comparability domain where adjustment for even a single confounder qualifies for a star. Important sources of bias inherent to retrospective designs, including selection bias, confounding by indication, and information bias, may not be fully captured by the NOS. Therefore, the uniformly high NOS scores should be interpreted with caution.

## 5. Conclusion

This meta-analysis suggests that elevated SIRI is associated with poorer OS and PFS in glioma patients, although this association was not consistently observed across all subgroups, particularly in European populations. These findings highlight the need for further validation with standardized cut-off values in diverse populations.

## Supporting information

S1 Table Literature search strategy. (DOCX)

S1 Fig Funnel plot (A) Overall survival; (B) progress free survival. (PDF)

S2 Fig Sensitivity analysis (A) Overall survival; (B) progress free survival. (PDF)

S1 Appendix PRISMA checklist. (DOCX)
