# Supplementary material for: Prognostic value of the systemic inflammation response index (SIRI) in glioma: A meta-analysis
Source: PLoS One. 2026 Nov 2;21(11):e0362113. doi: 10.1371/journal.pone.0362113 (PMC13726952; doi:10.1371/journal.pone.0362113)
Supplement: Supplementary file 1 [file pone.0362113.s002.pdf]

A

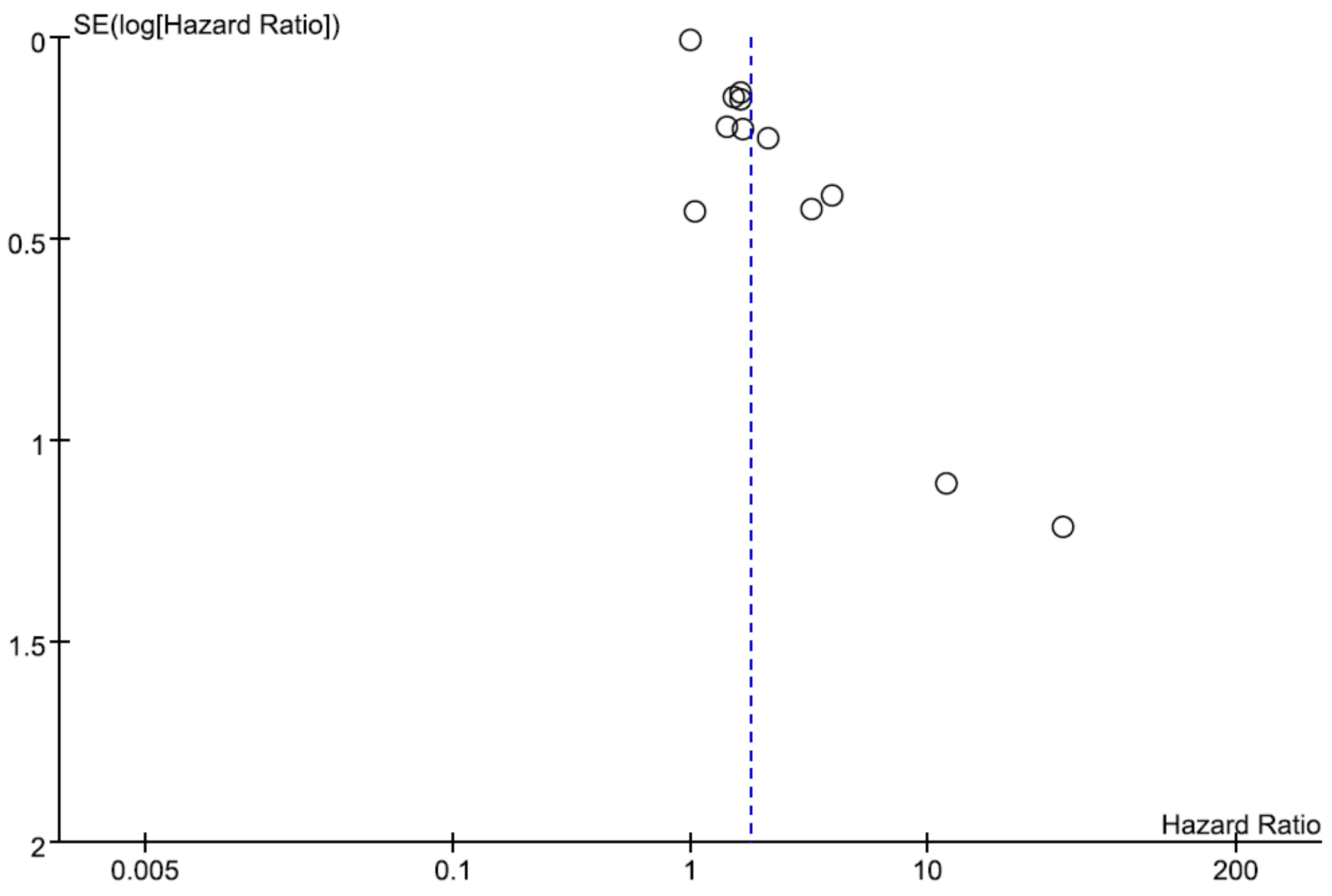
SE(log[Hazard Ratio])
0
0.5
1
1.5
2
0.005
0.1
1
10
200
Hazard Ratio

B

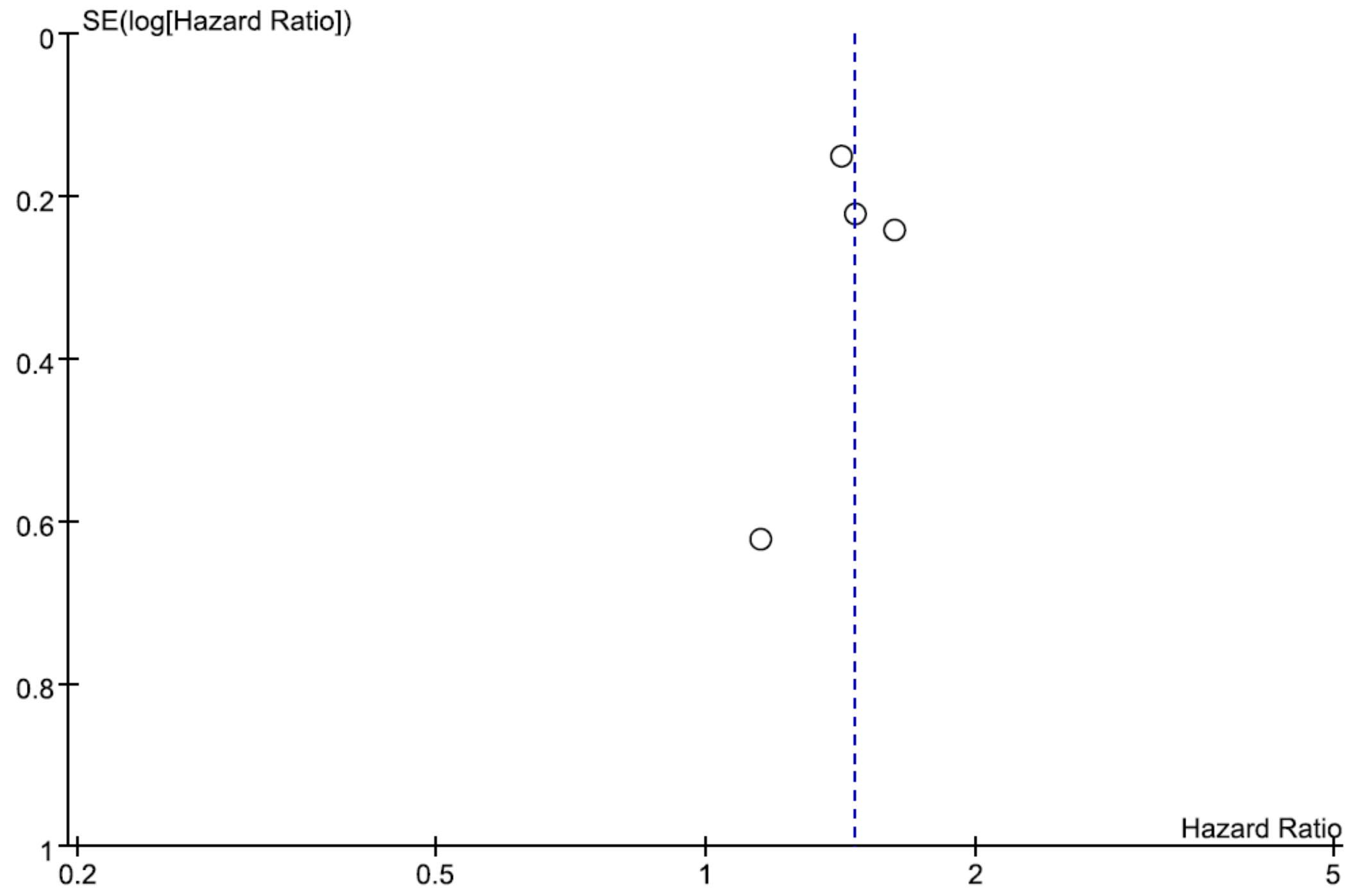
SE(log[Hazard Ratio])
0
0.2
0.4
0.6
0.8
1
0.2
0.5
1
2
5
Hazard Ratio
