# Supplementary material for: Prognostic value of the systemic inflammation response index (SIRI) in glioma: A meta-analysis
Source: PLoS One. 2026 Nov 2;21(11):e0362113. doi: 10.1371/journal.pone.0362113 (PMC13726952; doi:10.1371/journal.pone.0362113)
Supplement: Supplementary file 2 [file pone.0362113.s003.pdf]

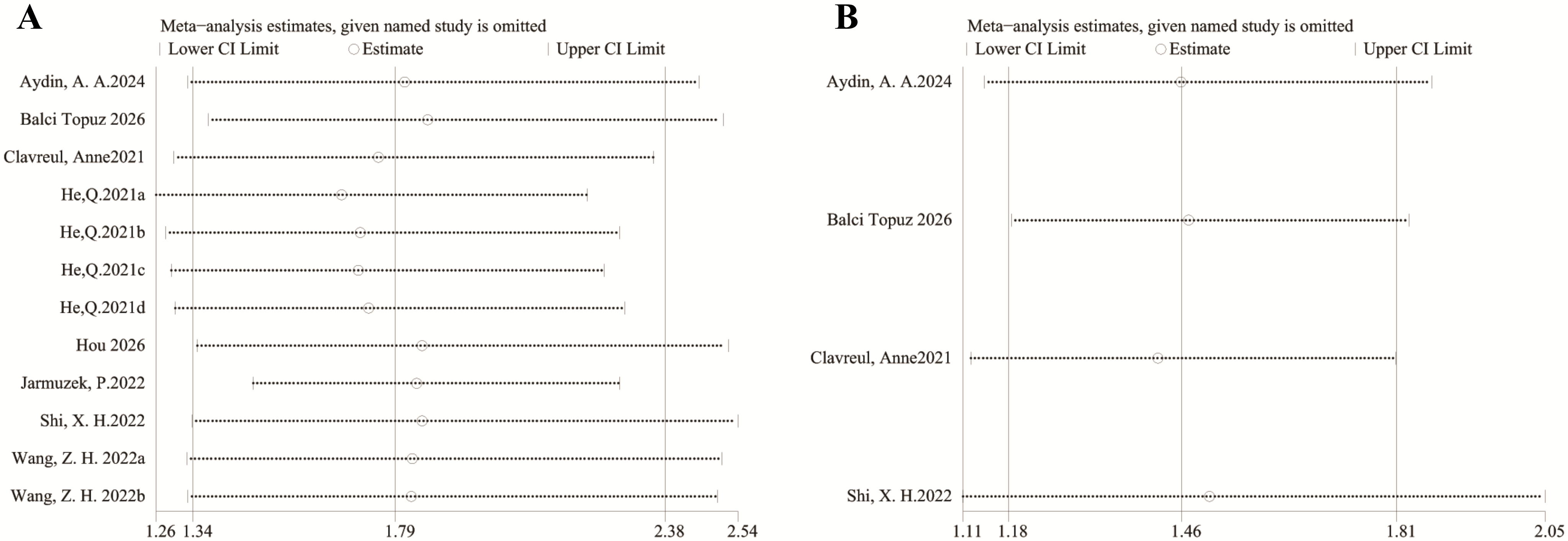
A
Meta−analysis estimates, given named study is omitted
Lower CI Limit
Estimate
Upper CI Limit
Aydin, A. A.2024
Balci Topuz 2026
Clavreul, Anne2021
He,Q.2021a
He,Q.2021b
He,Q.2021c
He,Q.2021d
Hou 2026
Jarmuzek, P.2022
Shi, X. H.2022
Wang, Z. H. 2022a
Wang, Z. H. 2022b
1.26
1.34
1.79
2.38
2.54
B
Meta−analysis estimates, given named study is omitted
Lower CI Limit
Estimate
Upper CI Limit
Aydin, A. A.2024
Balci Topuz 2026
Clavreul, Anne2021
Shi, X. H.2022
1.11
1.18
1.46
1.81
2.05
