# Supplementary material for: Prognostic value of the systemic inflammation response index (SIRI) in glioma: A meta-analysis
Source: PLoS One. 2026 Nov 2;21(11):e0362113. doi: 10.1371/journal.pone.0362113 (PMC13726952; doi:10.1371/journal.pone.0362113)
Supplement: Supplementary file 3 [file pone.0362113.s001.docx]

S1Table Searching strategy details

| Database | Strategy | Literature Number |
| --- | --- | --- |
| Pubmed | **("Glioma"[Mesh]) OR ((((((((Glioblastoma) OR (Gliomas)) OR (Glial Cell Tumors)) OR (Glial Cell Tumor)) OR (Mixed Glioma)) OR (Mixed Gliomas)) OR (Malignant Glioma)) OR (Malignant Gliomas))) AND ((systemic inflammation response index) OR (SIRI))** | 37 |
| Web of Science | **((Glioma) OR ((((((((Gliomas) OR (Glial Cell Tumors)) OR (Glial Cell Tumor)) OR (Mixed Glioma)) OR (Mixed Gliomas)) OR (Malignant Glioma)) OR (Malignant Gliomas)) OR (Glioblastoma))) AND ((systemic inflammation response index) OR (SIRI))** | 16 |
| Embase | **((Glioma) OR ((((((((Gliomas) OR (Glial Cell Tumors)) OR (Glial Cell Tumor)) OR (Mixed Glioma)) OR (Mixed Gliomas)) OR (Malignant Glioma)) OR (Malignant Gliomas)) OR (Glioblastoma))) AND ((systemic inflammation response index) OR (SIRI))** | 48 |
| Cochrane | **((Glioma) OR ((((((((Gliomas) OR (Glial Cell Tumors)) OR (Glial Cell Tumor)) OR (Mixed Glioma)) OR (Mixed Gliomas)) OR (Malignant Glioma)) OR (Malignant Gliomas)) OR (Glioblastoma))) AND ((systemic inflammation response index) OR (SIRI))** | 0 |
